# Dynamic Changes in Uterine NK Cell Subset Frequency and Function Over the Menstrual Cycle and Pregnancy

**DOI:** 10.3389/fimmu.2022.880438

**Published:** 2022-06-16

**Authors:** Emily M. Whettlock, Ee Von Woon, Antonia O. Cuff, Brendan Browne, Mark R. Johnson, Victoria Male

**Affiliations:** Department of Metabolism, Digestion and Reproduction, Institute of Reproductive and Developmental Biology, Imperial College London, London, United Kingdom

**Keywords:** NK cells, innate lymphocytes, decidua, endometrium, pregnancy, single-cell analysis

## Abstract

Uterine natural killer cells (uNK) play an important role in promoting successful pregnancy by regulating trophoblast invasion and spiral artery remodelling in the first trimester. Recently, single-cell RNA sequencing (scRNAseq) on first-trimester decidua showed that uNK can be divided into three subsets, which may have different roles in pregnancy. Here we present an integration of previously published scRNAseq datasets, together with novel flow cytometry data to interrogate the frequency, phenotype, and function of uNK1–3 in seven stages of the reproductive cycle (menstrual, proliferative, secretory phases of the menstrual cycle; first, second, and third trimester; and postpartum). We found that uNK1 and uNK2 peak in the first trimester, but by the third trimester, the majority of uNK are uNK3. All three subsets are most able to degranulate and produce cytokines during the secretory phase of the menstrual cycle and express KIR2D molecules, which allow them to interact with HLA-C expressed by placental extravillous trophoblast cells, at the highest frequency during the first trimester. Taken together, our findings suggest that uNK are particularly active and able to interact with placental cells at the time of implantation and that uNK1 and uNK2 may be particularly involved in these processes. Our findings are the first to establish how uNK frequency and function change dynamically across the healthy reproductive cycle. This serves as a platform from which the relationship between uNK function and impaired implantation and placentation can be investigated. This will have important implications for the study of subfertility, recurrent miscarriage, pre-eclampsia, and pre-term labour.

## Introduction

Uterine natural killer cells (uNK) are NK-like cells that are found in the lining of the uterus (called “decidua” in pregnancy and “endometrium” outside of pregnancy). They are distinct from peripheral blood NK (pNK) cells in both phenotype and function. Unlike pNK, uNK are only weakly cytotoxic and instead produce factors that are pro-angiogenic and that attract foetal derived placental cells called extravillous trophoblast (EVT) (1–6). uNK are most prominent in the first trimester of pregnancy, at which time they account for 70%–80% of immune cells in the decidua (7). Their prominence at the time of implantation, and their production of factors that are predicted to promote trophoblast invasion and spiral artery remodelling, indicates they are likely to have a role in placental implantation.

Further evidence for the role of uNK in implantation comes from their expression of high levels of killer-cell immunoglobulin-like receptors (KIRs), CD94/NKG2 (8, 9), and LILRB1 (9), which allows them to recognise the human leukocyte antigens (HLAs) expressed by EVT: HLA-C, HLA-E, and HLA-G, respectively (10–12). A number of immunogenetic studies have demonstrated that combinations of HLA and KIR that lead to lower activation are associated with disorders of insufficient implantation such as pre-eclampsia, foetal growth restriction, and recurrent miscarriage, suggesting that uNK activation *via* KIR is important for implantation (13–20), although it is important to note that not all studies have been able to find this association (21). The increased expression of KIRs by uNK around the time of implantation provides additional support for this idea (22).

CD107a staining is a proxy for degranulation, and previous studies have shown that this acts as a reliable measure of overall uNK activation (23). Uterine NK cells have been reported to produce IL-8 and granulocyte-macrophage colony-stimulating factor (GM-CSF): IL-8 is postulated to stimulate EVT invasion, whereas GM-CSF has been found to attract EVT (6, 23). On the other hand, the cytokines characteristically produced by pNK cells, TNFα and IFNγ, inhibit invasion of EVT cells and display increased production at later stages of early pregnancy, between 12 and 14 weeks (24, 25). However, there is some evidence that IFNγ may also be involved in spiral artery remodelling in early gestation (26).

Until recently, it was thought that uNK formed a single population, but single-cell RNA sequencing (scRNAseq) has now demonstrated three subpopulations of uNK in first-trimester decidua (27). These were originally called decidual NK (dNK) 1, 2, and 3, but they have now also been found in non-pregnant endometrium (28). Here, we call these subsets “uNK1,” “uNK2,” and “uNK3” in recognition of the finding that they are not confined to the decidua. uNK1 express higher levels of KIRs and LILRB1, indicating they may be specialised to communicate with EVT (27, 29). uNK2 and uNK3 produced more cytokines upon stimulation, indicating that their role may be immune defence (29). However, several questions remain open. Are these three subpopulations still present at the end of pregnancy? Do the subpopulations change in prominence and/or activity over the reproductive cycle? The answers to these questions could elucidate which subpopulations are important in implantation, parturition, and immune protection throughout the reproductive cycle.

Here, we show that the proportions of the uNK subpopulations remain stable through the menstrual cycle, but all three are more active and express higher levels of KIR around the time of implantation. uNK1 are more prominent in the first trimester of pregnancy, potentially indicating a requirement for this subset in the mediation of implantation, whereas uNK3 are the most prominent at the end of pregnancy. Overall, we outline how the three uNK subpopulations change in proportion, phenotype, and function throughout the reproductive cycle.

## Materials and Methods

### Primary Tissue

The collection of human tissue was approved by London—Chelsea Research Ethics Committee (study numbers: 10/H0801/45 and 11/LO/0971).

We examined tissues and collected data at seven stages of the reproductive cycle. In the menstrual cycle, there are three stages: menstrual (when the lining of the uterus is shed), proliferative (prior to ovulation), and secretory (after ovulation). Pregnancy is divided into three trimesters: first (1–12 weeks), second (12–28 weeks), and third (28 – 40 weeks). We also examined postpartum samples (up to 16 weeks post-delivery). During the menstrual cycle stages, the uterine tissue we examined is known as the endometrium. During pregnancy, this tissue undergoes a process called decidualisation and results in three tissues known as the decidua basalis (DB) (which lines the maternal side of the placenta), decidua parietalis (DP) (which lines the rest of the uterus), and decidua capsularis (which lines the embryo on the luminal side). When taking samples from first-trimester tissue, it is not possible to differentiate between the different decidual tissues. During the second trimester, the decidua capsularis fuses with the DP. When taking samples from third-trimester tissue, it is possible to dissect distinct samples from the DB and the DP.

A total of 29 endometrial samples were taken by Pipelle biopsy before insertion of an intrauterine device for contraception. Samples taken on a day of bleeding were assigned as a menstrual phase. Other samples were categorised into proliferative or secretory phase by date of last menstrual period and serum progesterone level: samples obtained before day 14 were assigned as proliferative phase and after day 14 as a secretory phase with retrospective confirmation by serum progesterone level, according to previously published reference range (30). We collected 8 menstrual, 7 proliferative, and 10 secretory phase samples and 4 samples from postpartum participants, up until 16 weeks postpartum.

A total of 10 decidual samples were taken from participants undergoing surgical management of elective termination of pregnancy between 6 and 13 weeks of pregnancy and 16 from participants undergoing elective caesarean sections, over 37 weeks of pregnancy and not in labour. For labouring data, a further 9 samples were taken from participants in the early stages of labour (1–3-cm cervical dilation and regular contractions) who had a caesarean section, and 9 samples were taken from participants after vaginal birth. Matched peripheral venous blood was obtained from all patients at the time of obtaining endometrial or decidual samples. Patient characteristics are summarised in **Supplementary Tables 1, 2**.

Lymphocytes were extracted from peripheral blood by layering onto Histopaque (Sigma-Aldrich, St. Louis, MO, USA), spinning down (700 ×*g*, 20 min, 21°C), and retrieving the interface, which was washed twice with Dulbecco’s Phosphate-Buffered Saline (PBS) (Life Technologies, Carlsbad, CA, USA) (500 ×*g*, 10 min, 4°C). Briefly, endometrial tissue was passed through a 100-µm cell strainer, pelleted (700 ×*g*, 10 min, 4°C), resuspended in Dulbecco’s PBS supplemented by 10% Foetal Calf Serum (FCS) (Sigma-Aldrich), passed through a 70-µm strainer, and layered on Histopaque as above.

For first-trimester samples, decidua compacta was extracted from products of conception and stirred for 20 min to remove blood before mincing with a scalpel followed by gentleMACS dissociation (Miltenyi, Bergisch Gladbach, Germany). Minced tissue was passed through a 75-µm sieve, pelleted (500 ×*g*, 10 min, 4°C), and resuspended in PBS/1% FCS before passing through a 100-µm strainer. The filtrate was layered on Histopaque as above.

For third-trimester DB samples, small sections were cut from the maternal side of the placenta and washed using a magnetic stirrer in Mg^2+^- and Ca^2+^-free PBS (Gibco, Grand Island, NY, USA) for 20 min. Blood clots, vessels, and placental tissue were physically removed, and cleaned decidual tissue was placed in new Mg^2+^- and Ca^2+^-free PBS. The tissue was spun (400 ×*g*, 5 mins 21°C), and PBS was removed. The tissue was resuspended in Accutase (Invitrogen, Carlsbad, CA, USA), mechanically digested in C tubes using a gentleMACS dissociater, and placed in a 37°C shaking water bath for 45 min. Minced tissue was passed through a 70-µm strainer and resuspended in PBS/1% FCS/2 mM EDTA. The filtrate was layered on Histopaque as above. For the third-trimester DP samples, 10 cm × 10 cm sections of the foetal membrane were dissected, and the decidua was removed using a cell scraper (Sarstedt, Nümbrecht, Germany). The tissue underwent enzymatic and mechanical digestion as described in the DB protocol.

Extracted lymphocytes were counted by light microscopy (Leica, Wetzlar, Germany) with a haemocytometer. A total of 0.2 × 10^6^ to 1 × 10^6^ cells per condition were allocated for phenotype and functional assessment.

### Stimulation With PMA/Ionomycin

A total of 21 endometrial, all first-trimester and all third-trimester samples were used for functional assessment. Endometrial lymphocytes were stimulated immediately after isolation, and decidual lymphocytes were stimulated after 12 to 20 h of rest at 37°C. Optimisation experiments showed no difference between cells stimulated fresh and after rest (**Supplementary Figure 1**).

For functional assessment, cells were suspended in Roswell Park Memorial Institute (RPMI) enriched with antibiotics, EDTA, and sodium pyruvate and divided into unstimulated and stimulated wells. Anti-CD107a BV605 (100 µl/ml), Brefeldin (10 µg/ml), and Monensin (2 µM/ml) were added to all wells and phorbol 12-myristate 13-acetate (PMA) (50 ng/ml) and ionomycin (1 µg/ml) into the stimulated wells only. Cells were incubated for 4 h at 37°C and then stained with antibodies. For third-trimester samples, cells were incubated for 6 h with anti-CD107a, PMA, and ionomycin, with Brefeldin and Monensin added 2 h into the incubation.

### Single-Cell RNA Sequencing Data Analysis

For the scRNAseq data, 5 stages of the reproductive cycle were examined: proliferative (n = 3), secretory (n = 3), first trimester (n = 5), second trimester (n = 1), and third trimester (term in labour (TNL) = 3). R (31) was used for the majority of the analysis. This included the use of the package Seurat (32), designed for analysis of scRNAseq data, and various data manipulation and visualisation packages (33–40).

A scRNAseq dataset from the non-pregnant uterus (available at www.reproductivecellatlas.org/) was converted from a Python format into an R Seurat object. The object was a subset of cells that had been classified as “Lymphoid” or “Myeloid” under “Cell.type” in the metadata.

A scRNAseq dataset from the first-trimester uterus (available at Array Express E-MTAB-6701) was converted from.txt into an R seurat object. The object was a subset of cells that had been designated an immune cell type, e.g., “dNK1” under “Annotation,” in the metadata. Cells originating from the placenta or blood were removed, so only decidual cells remained. Data from one donor were removed from the analysis due to their NK cells clustering independently of all other NK cells.

The scRNAseq dataset (dbGaP phs001886.v1.p1, reanalysed with permission of the NIH, project ID 26528) contained samples from one second-trimester accreta case, which is therefore pathological, and 9 third-trimester participants. Both datasets were filtered, aligned, and quantified using Cell Ranger software (version 5.0.1, 10x Genomics). h19 was used as a human genome reference. Downstream analyses were performed using the R package Seurat (version 4.0.2) (32). Cells with fewer than 200 genes and genes that were expressed in less than 10 cells were removed. Furthermore, cells where the gene content was greater than 10% mitochondrial genes were removed. Clusters were identified using “FindClusters” algorithm. The “FindAllMarkers” algorithm was used to identify the immune clusters and subset the object to immune cells. Cells from placental tissue were removed. TIL and PTL samples were not included in the analysis observing uNK across the reproductive cycle. PTL samples were not included in the term labouring analysis.

The four datasets were integrated based on a previously published workflow (41). Clusters that appeared to be non-immune cells were removed, and the remaining cells were reanalysed using the same workflow. The integrated dataset contained 2,180 cells from non-pregnant endometrium, 18,243 cells from first-trimester decidua, 3,900 from second-trimester decidua, and 6,077 from third-trimester decidua. The algorithm “FindConservedMarkers” was used to identify the clusters uNK1, uNK2, and uNK3. This was confirmed by the metadata column “annotation” from the first-trimester dataset. For visualisation of clusters and gene expression across the reproductive cycle, each dataset was downsampled so that the total number of cells displayed was equal in each dataset.

### Flow Cytometry

The following anti-human antibodies were used: anti-CD56 Brilliant Violet (BV) 650 (clone NCAM 16.2, BD Biosciences, San Jose, CA, USA), anti-CD39 BV421 (clone A1, BioLegend, San Diego, CA, USA), anti-CD3 BV711 (clone SK7, BioLegend), anti-CD103 BV785 (clone Ber-ACT8, BioLegend), anti-CD16 Alexa Fluor(AF)700 (clone 3G8, BioLegend), anti-CD9 phycoerythrin(PE)/Dazzle 594 (clone HI9a, BioLegend), anti-CD49a PE/Cy7 (clone TS2/7, BioLegend), anti-CD45 allophycocyanin (APC) (clone HI30, BioLegend), anti-CD94 PE (clone HP-3D9, BD Biosciences), anti-CD158a/h (KIR2DL1/DS1) VioBright 515 (clone REA1010, Miltenyi Biotec, Bergisch Gladbach, Germany), anti-CD158b (KIR2DL2/DL3) APC vio 770 (clone REA 1006, Miltenyi Biotec), CD85j (ILT2 or CD94) Peridinin chlorophyll protein (PerCP)-eFluor 710 (clone HP-F1, Thermo Fisher Scientific, Waltham, MA, USA) and anti-CD107a BV605 (clone H4A3, BioLegend) for surface antigens, and anti-IL-8 PE (clone G265-8, BD Biosciences), anti-IFN-γ APCvio770 (clone REA600, Miltenyi Biotec), anti-GM-CSF PERCP/Cyanine 5.5 (clone BVD2-21C11, BioLegend), and anti-TNFα fluorescein isothiocyanate (FITC) (clone MAb11, BioLegend) for intracellular staining.

Cells were first incubated with fixable viability dye (Live/Dead Fixable Aqua Dead Cell stain kit, Life Technologies) (15 min, 4°C) followed by incubation with surface antibodies (15 min, 4°C). For intracellular staining, human FoxP3 buffer (BD Biosciences) was used according to the manufacturer’s instructions before staining with intracellular antibodies (30 min, 4°C). For third-trimester samples, fixable viability dye was included with the surface staining antibodies (20 min, RT) and intracellular staining using the Cytofix/Cytoperm kit (BD Biosciences) according to the manufacturer’s instructions. Excess antibodies were washed off (5 min, 500 ×*g*, 4°C) between each incubation and twice after the final incubation with intracellular antibodies.

### Statistical Analysis

Data were acquired on a BD Fortessa and analysed using FlowJo (Tree Star, Ashland, OR, USA). Application settings were used to ensure reproducible results. Statistical analysis was performed using PRISM (GraphPad Software Inc.). Data were assessed for normality using Shapiro–Wilk tests to determine whether a parametric or a non-parametric statistical test was appropriate. The appropriate statistical test was used to compare subsets as specified in figure legends. p < 0.05 was considered significant.

## Results

### uNK1, uNK2, and uNK3 Are Present Throughout the Human Reproductive Cycle and Vary in Frequency

**Figure 1:**
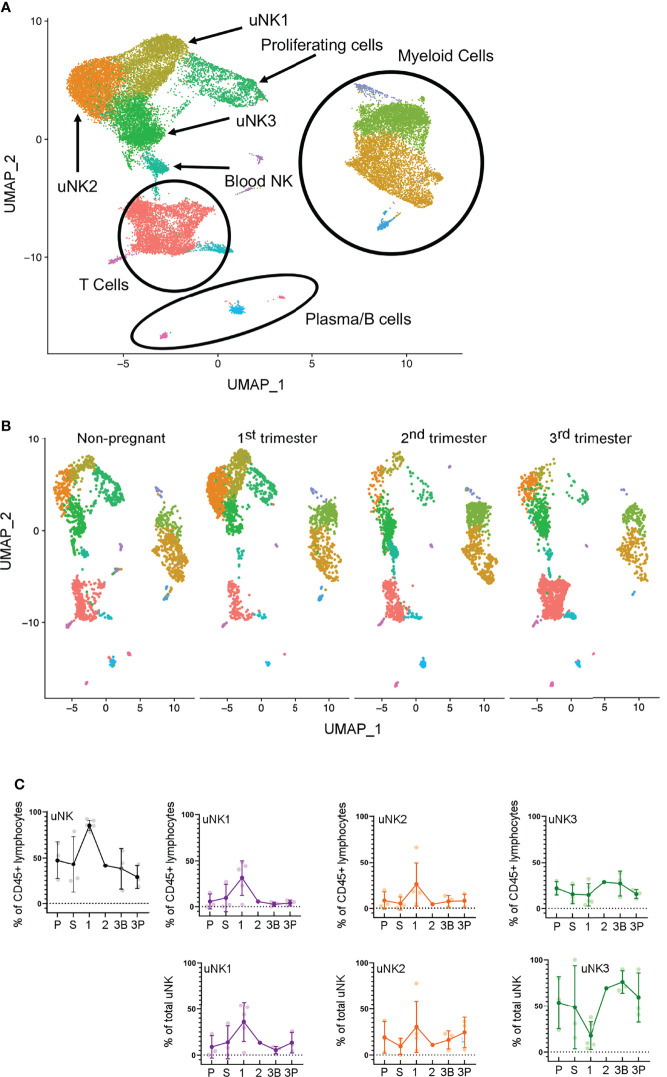
uNK1, uNK2, and uNK3 are present throughout the human reproductive cycle, by scRNAseq. **(A)** Integrated immune cells from non-pregnant endometrium, and first-, second-, and third-trimester decidua, visualised by Uniform Manifold Approximation and Projection (UMAP). Colours are indicative of clusters and are identified with appropriate immune cell types. n = 12 (non-pregnant), n = 5 (1T), n = 1 (2T), n = 3 (3T). uNK, uterine natural killer. **(B)** Immune cells from each of the four stages in the reproductive cycle subset to 2,200 cells. Immune cells separated by stage and then visualised by UMAP. Colours are indicative of clusters. **(C)** Using the scRNAseq dataset, graphs show frequency of total NK from CD45+ lymphocytes and then frequency of each uNK subset (uNK1, uNK2, and uNK3) as both a proportion of CD45+ lymphocytes and a proportion of total uNK cells. Means and SDs are shown for n = 3 (proliferative), n = 3 (secretory) n = 5 (first trimester), n = 1 (second trimester), n = 3 (third-trimester decidua basalis), and n = 3 (third-trimester decidua parietalis). P, proliferative phase; S, secretory phase; 1, first trimester; 2, second trimester; 3B, third-trimester decidua basalis; 3P, third-trimester decidua parietalis.

scRNAseq analysis has previously identified that three subpopulations of uNK, uNK1, uNK2, and uNK3, are present in first-trimester decidua (27) and non-pregnant endometrium (28). Previous analysis of scRNAseq data from third-trimester decidua identified only a single cluster within the uNK population (42), and our reanalysis of the third-trimester dataset alone confirmed this. However, when the third-trimester data were integrated with data from the non-pregnant uterus, first- and second-trimester, the third-trimester uNK cells did form three clusters (**Figures 1A, B**).

**Figure 2:**
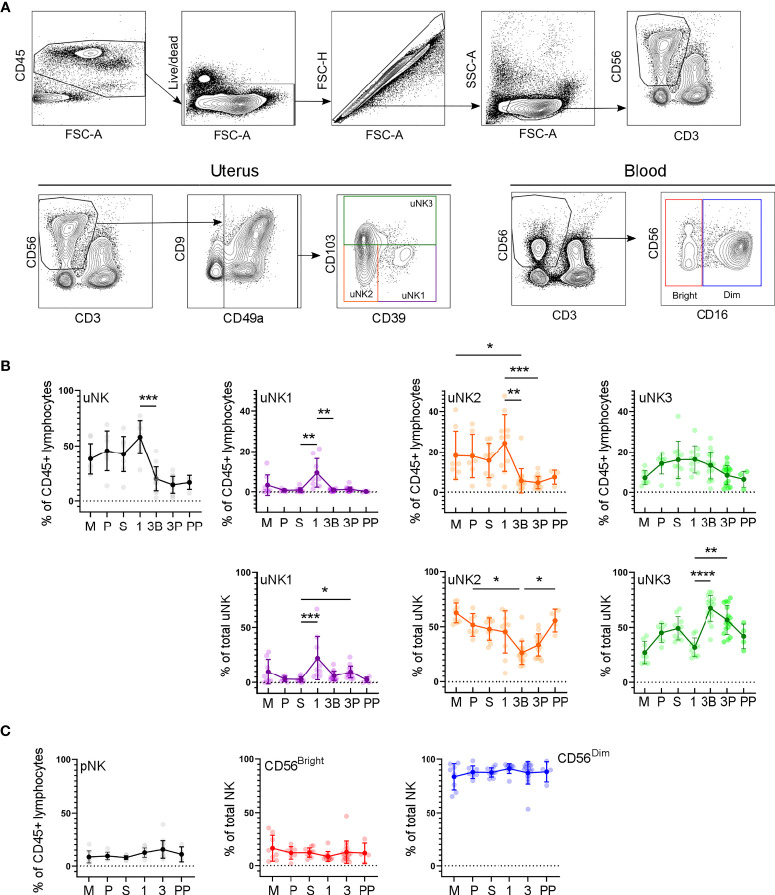
uNK1, -2 and -3 are present throughout the human reproductive cycle, by flow cytometry. **(A)** FACs gating strategy used to identify three uNK subsets and pNK (representative example shown). Coloured boxes in final plot indicate colour used for that subset in subsequent graphs. **(B)** Using flow cytometry data, graphs show frequency of total NK from CD45+ lymphocytes and then frequency of each uNK subset (uNK1, -2 -3) both as a proportion of CD45+ lymphocytes and proportion of total uNK cells. Means and standard deviations are shown for n = 8 (menstrual), n = 7 (proliferative), n = 10 (secretory) n= 10 (first trimester), n= 16 (third trimester decidua basalis), n = 16 (third trimester decidua parietalis), n = 4 (postpartum). Statistical testing was done using Kruskal Wallis with a post-hoc Dunn test *p < 0.05, **p < 0.01, ***p < 0.001, ****p< 0.0001. **(C)** Using flow cytometry data, graphs show frequency of total NK from CD45+ lymphocytes and then frequency of each pNK subset (CD56^Bright^ and CD56^Dim^) as a proportion of total pNK. Sample numbers for each group are the same as B. M, menstrual phase; P, proliferative phase; S, secretory phase; 1, first trimester; 3B, third trimester decidua basalis; 3P, third trimester decidua parietalis; PP, post-partum.

In the first trimester, uNK can be distinguished from circulating NK cells by their expression of CD49a and CD9; the subsets are then defined by their expression of CD39 and CD103 (27). We confirmed the presence of CD49a+ uNK in the endometrium and in the first- and third-trimester decidua and that the three subpopulations uNK1, uNK2, and uNK3 can be identified using CD39 and CD103 (**Figure 2A**). However, in third-trimester samples, there was a significant CD49a+CD9− population. A comparison of CD49a+CD9+ and CD49a+ CD9− detected no phenotypic differences between these two populations, suggesting that CD49a alone can be used to identify uNK cells in the third trimester (**Supplementary Figure 2**). For consistency of gating strategy, we therefore also identified uNK cells by their expression of CD49a alone in the endometrial, first-trimester, and postpartum samples.

In line with previous reports (7), we observed a peak in total uNK, as a proportion of total CD45+ lymphocytes, in first-trimester pregnancy by both scRNAseq and flow cytometry (**Figures 1C**, **2B**). We observed a similar proportion of total uNK in the proliferative and secretory phases (**Figures 1C**, **2C**). Additionally, we analysed CD56+CD49a− cells (which represent pNK cells) as a proportion of total CD56+ NK cells in endometrium/decidua and found this to be significantly higher in third- compared to first-trimester decidua; however, no difference was noted through the menstrual cycle [median (interquartile range (IQR)) for menstrual, 27.7 (38.1); proliferative, 23.8 (20.7); secretory, 22.3 (19.1); first trimester, 8.1 (5.0); third trimester DB 26.7 (12.4); third trimester DP 38.2 (22.6), postpartum, 13.6 (12.3)]. This highlights the importance of using tissue-specific immune cell markers, particularly when examining third-trimester decidua.

Next, we examined uNK1, uNK2, and uNK3 frequency expressed as a proportion of either total CD45+ lymphocytes or total uNK. We observed an increase in the frequency of uNK1 when transitioning from the secretory phase to first-trimester pregnancy, but this was not sustained into the third trimester. This observation applied to uNK1 as a percentage of both CD45+ lymphocytes and the percentage of total NK cells (**Figures 1C**, **2B**), and the change was significant when measured by flow cytometry.

The variation of uNK2 frequency was similar to that observed for uNK1, with a peak in the first trimester observed by scRNAseq, and flow cytometry when the frequency was measured as a percentage of CD45+ lymphocytes (**Figures 1C**, **2B**). For the latter, uNK2 was significantly higher in the first trimester, compared to the third. Further, there was an upward trend of uNK2 when transitioning from third-trimester decidua to postpartum endometrium when measured as a proportion of total NK (**Figure 2C**).

For uNK3, there was no change in frequency through the menstrual cycle. When measured as a percentage of CD45+ lymphocytes, there was a reduction in uNK3 in third-trimester DP, compared to both first-trimester decidua and third-trimester DB. This was significant when measured by flow cytometry. When measuring uNK3 as a percentage of total uNK, there was a dip in the first trimester and a peak in both types of the third-trimester decidua. This was significant when measured by flow cytometry. The discrepancy between the proportions when expressed as a percentage of CD45+ lymphocytes or total uNK cells is likely due to the change in frequency of total uNK, as a proportion of CD45+ lymphocytes.

Within the third-trimester decidua, the uNK2 population appeared greater as a proportion of total uNK in the DP compared to the DB in both scRNAseq and flow cytometry, although this did not reach significance for either (**Figure 1C**, **2B**). The uNK3 population appeared greater in the DB, compared to the DP, which was significant when measured by flow cytometry as a percentage of CD45+ lymphocytes (**Figure 1C**, **2B**).

### Peripheral Blood NK Cell Frequency Does Not Vary Over the Reproductive Cycle or Correlate With uNK Frequency

We also examined CD56^Bright^ and CD56^Dim^ NK cells in matched peripheral blood, using a conventional gating strategy to identify these populations (**Figure 2C**). Unlike uNK, there was no variation in total CD56+ pNK, CD56^Bright^, or CD56^Dim^ in peripheral blood when transitioning through different phases of the reproductive cycle. Furthermore, there was no significant correlation in levels of pNK and uNK subsets when expressed as either a proportion of CD45+ live lymphocytes or total NK cells (**Supplementary Figure 3**).

### uNK Subsets Upregulate KIR and LILRB1 During Transition From Non-Pregnant Endometrium to First-Trimester Decidua

**Figure 3:**
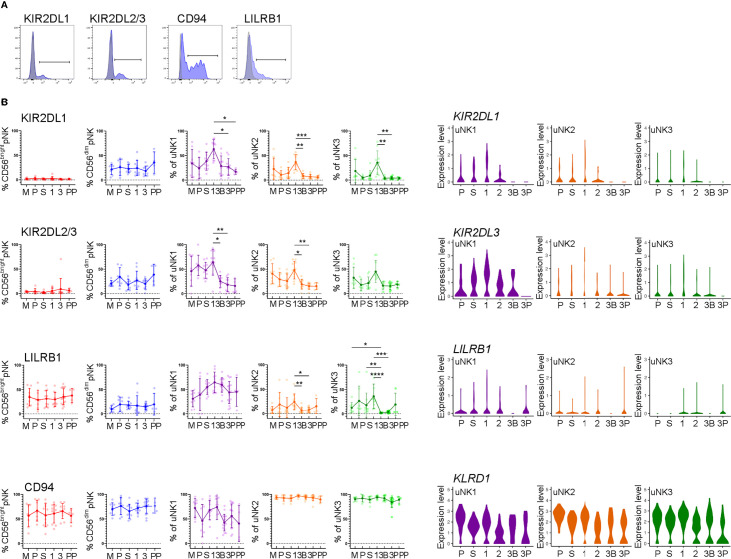
uNK upregulate expression of KIR and LILRB1 in first trimester. **(A)** Uterine and peripheral NK cells taken at different stages of the reproductive cycle were freshly stained for phenotypic markers. Representative staining from CD56dim pNK is shown in blue alongside FMO controls in grey. **(B)** Graphs showing frequencies of KIR2DL1, KIR2DL2/3, LILRB1 and CD94 on pNK and uNK subsets. Means and standard deviations are shown for n = 8 (menstrual), n = 7 (proliferative), n = 10 (secretory) n= 10 (first trimester), n= 16 (third trimester decidua basalis), n = 16 (third trimester decidua parietalis), n = 4 (postpartum). Statistical testing was done using Kruskal Wallis with a post-hoc Dunn test *p < 0.05, **p < 0.01, ***p < 0.001, ****p< 0.0001. **(C)** Violin plots showing corresponding mRNA expression in uNK subsets over the reproductive cycle as determined by scRNAseq. M, menstrual phase; P, proliferative phase; S, secretory phase; 1, first trimester; 2, second trimester; 3, third trimester; 3B, third trimester decidua basalis; 3P, third trimester decidua parietalis.

We next examined uNK expression of receptors that interact with trophoblast cells: KIR2DL1 and KIR2DL2/3 recognise HLA-C, LILRB1 recognises HLA-G, and CD94 recognises HLA-E (**Figure 3A**) (43).

In line with earlier findings on first-trimester uNK (27, 29), we observed that uNK1 expressed higher levels of KIR than uNK2 and uNK3 (**Figures 3B, C**). We also found that all three uNK subsets expressed increased KIR in the first trimester of pregnancy, compared to non-pregnant endometrium and third-trimester decidua (**Figures 3B, C**). Similar to KIR, LILRB1 protein expression peaked in the first trimester, although this was only statistically significant in uNK2 and uNK3 (**Figure 3B**). *LILRB1* transcript expression followed a similar trend, although in contrast to our findings at the protein level, *LILRB1* mRNA was not detectable in the DB (**Figure 3C**). At the transcript and protein levels, CD94 was expressed at a higher level on uNK2 and -3 compared to uNK1 (**Figures 3B, C**). There was a slight reduction in CD94 transcript (*KLRD1*) towards the end of pregnancy, but this was not observed at the protein level (**Figures 3B, C**).

In line with our finding that pNK did not change in frequency over the reproductive cycle, examination of NK cells from matched blood showed no change in the frequency at which KIR, LILRB1, and CD94 are expressed in these cells (**Figure 3B**).

### uNK Are the Most Active at the Time of Implantation

**Figure 4:**
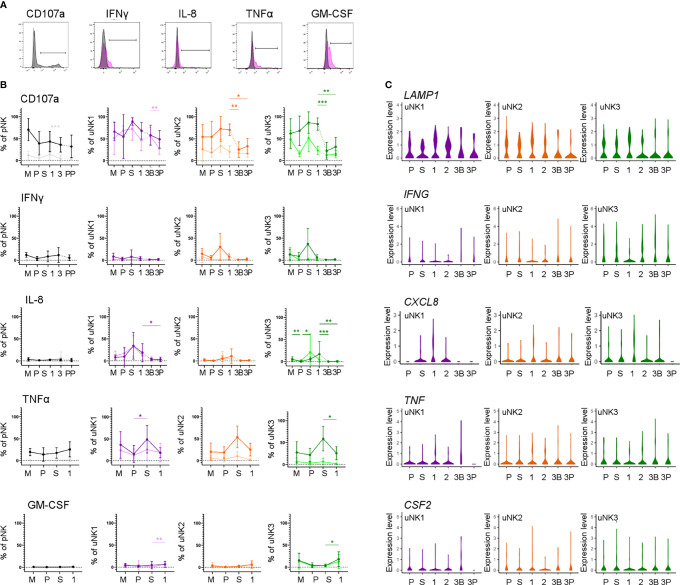
uNK are most active at the time of implantation. **(A)** Uterine and peripheral NK cells taken at different stages of the reproductive cycle were cultured with or without PMA and ionomycin stimulation and assessed for degranulation (CD107a) and production of IFNγ, IL-8, TNFα and GM-CSF. Representative staining of CD107a on total pNK is shown in black, alongside FMO controls in light grey. Representative staining from secretory phase uNK1 is shown for the cytokines in pink, alongside internal negative controls in grey. The positive gates were set by reference to FMO controls. **(B)** Graphs showing frequencies of CD107a, IFNγ, IL-8, TNFα and GM-CSF on pNK and uNK subsets. Unstimulated cells are represented by lighter lines and stimulated cells by darker lines. Means and standard deviations are shown for n = 5 (menstrual), n = 6 (proliferative), n = 10 (secretory) n= 10 (first trimester), n= 16 (third trimester decidua basalis), n = 16 (third trimester decidua parietalis). Statistical testing was done using Kruskal Wallis with a post-hoc Dunn test *p < 0.05, **p < 0.01, ***p < 0.001, and ****p < 0.0001. **(C)** Violin plots showing corresponding mRNA expression in uNK subsets over the reproductive cycle as determined by scRNAseq. M, menstrual phase; P, proliferative phase; S, secretory phase; 1, first trimester; 2, second trimester; 3, third trimester; 3B, third trimester decidua basalis; 3P, third trimester decidua parietalis.

We next assessed functional responses with and without stimulation with PMA and ionomycin (**Figure 4A**). Degranulation in unstimulated conditions declined during the proliferative phase, slightly in uNK2, and significantly in uNK3, compared to the other two phases of the menstrual cycle (**Figure 4B**). At the end of pregnancy, degranulation was significantly lower in the third-trimester DP compared to DB in uNK1, but this was not replicated in the other subsets (**Figure 4B**). In stimulated cells, there was a reduction in degranulation in uNK2 and uNK3 in both third-trimester decidua compared to first-trimester decidua (**Figure 4B**).

For TNFα, IFNγ, and IL-8, we observed peaks in cytokine production across all stimulated uNK subsets during the secretory phase, compared to the proliferative phase and first trimester, although this did not reach statistical significance in all cases (**Figure 4B**). For IL-8 in uNK3, this peak was maintained into first-trimester pregnancy. This trend was also present in unstimulated cells for IL-8 (**Figure 4B**).

Third-trimester uNK produced less cytokine than first-trimester uNK, although this only reached significance in IL-8 production from uNK3. This reduction in cytokine production through pregnancy was also seen at the mRNA level for IL-8 (**Figure 4C**). In contrast, GM-CSF protein production was consistently low in the menstrual cycle, including the secretory phase, but increased significantly in the first trimester of pregnancy in unstimulated uNK1 and stimulated uNK3 (**Figure 4B**).

For examination of NK cells from matched peripheral blood, data from CD56^Bright^ and ^Dim^ NK cells are shown together due to downregulation of CD16 after stimulation. Aside from a significant decline in CD107a expression in unstimulated cells when transitioning from the secretory phase to first-trimester pregnancy, there was no distinct trend in either stimulated or unstimulated cells (**Figure 4B**).

The mRNA expression of other NK cell proteins of interest across the reproductive cycle, such as granzymes, that were not included in the flow cytometry panel, can be seen in **Supplementary Figures 4**, **5**.

### uNK Phenotype and Function Do Not Change in Labour

**Figure 5:**
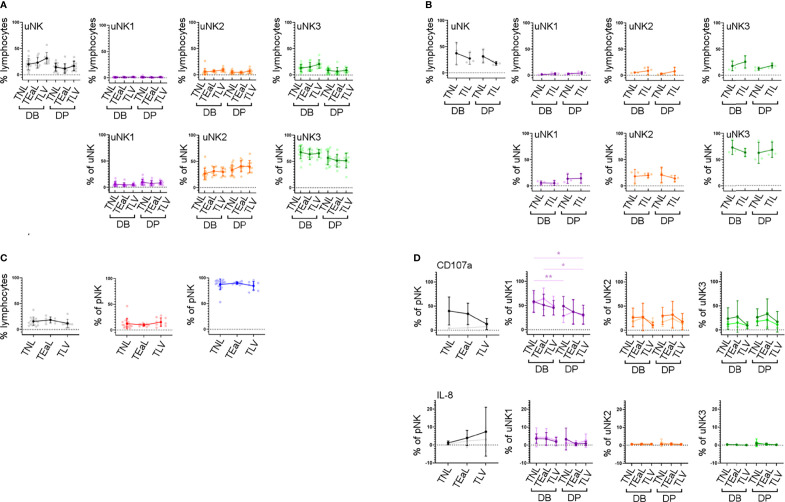
uNK phenotype and function do not change in labour. **(A)** The FACs gating strategy shown in **Figure 2A** was used to identify the three uNK subsets. Graphs show frequency of total NK from CD45+ lymphocytes and then frequency of each uNK subset (uNK1, uNK2, and uNK3) as both a proportion of CD45+ lymphocytes and proportion of total uNK cells. Graphs are divided to show results from both decidua basalis and decidua parietalis. Means and SDs are shown for n = 16 (TNL), n = 9 (TEaL), and n = 9 (TLV). Statistical testing was done using Kruskal–Wallis with a *post-hoc* Dunn test: * p < 0.05, and ** p < 0.01. **(B)** Using the scRNAseq dataset, graphs show frequency of total NK from CD45+ lymphocytes and then frequency of each uNK subset (uNK1, uNK2, and uNK3) as both a proportion of CD45+ lymphocytes and proportion of total uNK cells. Graphs are divided to show results from both decidua basalis and decidua parietalis. Means and SDs are shown for n = 3 (TNL) and n = 3 (TIL). **(C)** The FACs gating strategy shown in **Figure 2C** was used to identify the two pNK subsets. Using flow cytometry data, graphs show frequency of total NK from CD45+ lymphocytes and then frequency of each pNK subset (CD56^Bright^ and CD56^Dim^) from total NK. Sample numbers for each group are the same as in panel **(A, D)** Uterine and peripheral NK cells were cultured as described in **Figure 4A**. Graphs showing frequencies of CD107a and IL-8 on pNK and uNK subsets. Unstimulated cells are represented by lighter lines and stimulated cells by darker lines. n, numbers for each group are the same as in panel **(A)** Statistical testing was done using Kruskal–Wallis with a *post-hoc* Dunn test: * p < 0.05, and ** p < 0.01. DB, decidua basalis; DP, decidua parietalis; TNL, term non-labouring; TEaL, term early labouring; TLV, term vaginal birth; TIL, term in labour.

In line with previous findings (44, 45), we did not observe any change in the proportion of total uNK in labouring compared to non-labouring decidua (**Figure 5B**). The proportion of uNK in non-labouring decidua was lower in the DP compared to the DB (**Figure 5B**). We next examined the uNK subpopulations in non-labour compared to early labour and established labour samples. We observed no change in the frequency of any of the uNK or pNK subsets across the spectrum of these samples (**Figures 5A, B**). The receptors examined were also stably expressed during labour (**Supplementary Figure 6**), an observation that suggests that EVT cross-talk with uNK is not a major participant in labour. Similarly, for those markers examined, the function of uNK and pNK subsets remains stable during labour, although we did observe that, regardless of labouring state, the uNK1 population in the DB is significantly more active than the population in the DP (**Figure 5** and **Supplementary Figure 7**). This is supported by the low number of differentially expressed genes across all three uNK subsets in the scRNAseq data compared with non-labour to labour samples (**Supplementary Table 3**).

## Discussion

To our knowledge, this is the first study to track the three uNK subpopulations throughout the reproductive cycle, examining their frequency, phenotypes, and functions, although one limitation is the small sample size in several of the groups, which should be taken into consideration during interpretation of these results. In line with previous studies, we found that the total uNK population peaked during the first trimester of pregnancy (7, 46). We discovered that uNK1 and uNK2 peak in this period but that uNK3 peaks towards the end of pregnancy. This aligns with a recent report that KIR+CD39+ uNK (mostly representing uNK1) and KIR+CD39− (mostly representing uNK2) increase in frequency towards the end of the menstrual cycle and remain elevated in early pregnancy (47). Both these reports support the proposal that uNK1 communicate with EVTs in early pregnancy (27, 29) but may also point to a role for uNK2 in this process.

Although previous studies have agreed that uNK fluctuate over the menstrual cycle, there has been some disagreement about precisely how. Studies using immunohistochemistry showed an increase in numbers of CD56+ cells in the secretory compared to the proliferative phase (48–51). This finding was borne out by one study that demonstrated a significant increase in CD3−CD56+ NK cells as a proportion of CD45+ cells in the early secretory compared to the late proliferative stage by flow cytometry (52), but two others were unable to detect this difference (49, 53). In our study, which used CD49a to remove pNK from the analysis thereby giving a true reflection of tissue-resident uNK, we were also unable to detect a change in the proportion of uNK through the menstrual cycle. However, a possible limitation is that we did not classify our secretory phase samples into early, mid, and late, which may have limited our ability to detect differences observed in studies with sufficient samples to use this more rigorous classification.

Compared to pNK, uNK cells are biased towards the expression of KIR2D expression: one previous study using tissue samples found this bias only in first-trimester decidua (22), whereas a more recent study also detected this bias in uNK isolated from menstrual blood, and therefore originating in the non-pregnant uterus (54). Here, we find a clear peak in the expression of KIR2D expression specifically in the first trimester, among all three uNK subsets. A similar trend was seen for LILRB1. This suggests that all uNK subpopulations increase their ability to recognise EVT, *via* HLA-C and HLA-G, in the first trimester. In comparison, CD94 expression was higher on uNK2 and uNK3 subsets compared to uNK1. This is in contrast with previous findings (29). However, the earlier study measured marker intensity on recovered cryopreserved cells, whereas we report a percentage of CD94+ fresh cells. It is possible that the freezing process preferentially killed CD94− uNK1 cells or that uNK1 have a lower percentage of cells expressing CD94 but a higher expression per cell: either of these could explain the disparity in our results. Other markers that may be of interest, which were not examined in our study, are KIR2DL4, which has been suggested to be a receptor for HLA-G and HLA-F (55, 56).

By examining degranulation as a proxy for uNK cell activation (23), we found that uNK were typically most active around the time of implantation, in the secretory phase of the menstrual cycle and the first trimester of pregnancy. This is in line with previous findings that first-trimester uNK are more able to degranulate in response to HCMV-infected targets than those at term, although the same study found that, following IL-15 stimulation, term uNK are better able to degranulate in response to PMA and ionomycin than first-trimester cells (9). Without stimulation, uNK produce little IFNγ, IL-8, TNFα, and GM-CSF. However, after stimulation with PMA and ionomycin, uNK have the highest ability to produce most of these cytokines during the secretory phase. This may be in line with a previous report suggesting that endometrial NK cells are more transcriptionally active than decidual NK cells (57). The exception was GM-CSF, whose production peaks in the first trimester. Previous studies suggest that the ability of uNK to produce growth factors and cytokines evolves as pregnancy progresses, with production predominantly of pro-angiogenic and growth factors at 8–10 weeks, and predominantly cytokines at 12–14 weeks (58). We did not collect sufficient first-trimester samples to undertake an analysis stratified by gestational age, but nonetheless, our finding that the timing of maximum activation coincides with the window of implantation is interesting because it suggests that uNK may have a role in coordinating successful implantation.

Cumulative evidence indicates an important physiological role for uNK in first-trimester pregnancy, but there is conflicting evidence on their role in reproductive failure. Our findings and those of others (27, 29) point to uNK1 as the uNK subset more likely to mediate placental implantation in early pregnancy. Future studies focusing specifically on this subset may be able to elucidate differences that were previously masked due to the examination of uNK as a bulk population. A recent study using scRNAseq suggests that there is a reduction of uNK1 in pathological pregnancies (59); however, these findings should be interpreted with caution because the pathological samples were collected after pregnancy loss, making it difficult to discern if changes seen in immune cells are a cause or an effect of pregnancy loss, due to inflammatory changes that typically occur after foetal demise. To overcome this, future studies could interrogate uNK during the window of implantation (60) or from elective termination of pregnancy samples stratified to low and high risk by uterine artery Doppler ultrasound, which has high specificity in predicting the risk of pre-eclampsia and intrauterine growth restriction (61).

In the third trimester, we found a greater proportion of uNK in the DB compared to DP. This is in contrast to a previous report that found a higher frequency of CD56B^right^ NK cells in the parietalis than in the basalis (62). However, the lack of tissue-specific markers in this earlier study means that it is difficult to be sure if these all represent uNK. In both decidual tissues in the third trimester, the majority of uNK cells are uNK3, which express low levels of KIRs and LILRB1. This could suggest that, in contrast to early pregnancy, the major role of uNK in late pregnancy does not involve interactions with EVTs. Similarly, the expression of the functional markers we examined was lower in the third trimester. This could indicate that, if these cells have a role at the end of pregnancy, it is *via* a different mechanism of action. The resemblance of uNK3 to intra-epithelial ILC1 (29) could suggest that their role is in mucosal homeostasis, and this is likely to be important throughout pregnancy, in line with the relative constancy of uNK3, compared to uNK1 and uNK2. Intriguingly, by scRNAseq, uNK3 were the subset that differed the most, transcriptionally, between non-labouring and labouring states, suggesting they may have a role in labour that is yet to be defined. It would be interesting to do a broader analysis of the potential functions of these cells or examine their roles in pathological cases such as pre-eclampsia or preterm birth.

In conclusion, we show here how uNK subset number, expression of receptors, and function change dynamically across the healthy reproductive cycle. This provides evidence of their physiological role in implantation but will also provide an important platform from which the relationship between uNK function and pathologies of pregnancy associated with impaired implantation and placentation can be investigated.

## Data Availability Statement

The datasets presented in this study can be found in online repositories. The names of the repository/repositories and accession number(s) can be found below: www.reproductivecellatlas.org/, https://cellgeni.cog.sanger.ac.uk/vento/reproductivecellatlas/endometrium_all.h5ad; https://www.ncbi.nlm.nih.gov/projects/gap/cgi-bin/study.cgi?study_id=phs001886.v1.p1; and https://osf.io/wkxyz/, https://osf.io/wkxyz/. Code is available from https://github.com/ewhettlock/reproductive_cycle.

## Ethics Statement

The collection of human tissue was approved by London—Chelsea Research Ethics Committee (study numbers: 10/H0801/45 and 11/LO/0971). The patients/participants provided their written informed consent to participate in this study.

## Author Contributions

EMW, EVW, and VM designed the study, analysed the results, and wrote the manuscript. EMW, EVW, and AC carried out the experiments. EVW, BB, and MJ obtained patients’ consent and collected the clinical samples. All others contributed to editing the manuscript. All authors listed have made a substantial, direct, and intellectual contribution to the work and approved it for publication.

## Funding

This study was funded by Borne. Study phs001886.v1.p1 was, in part, supported by the Perinatology Research Branch, Division of Obstetrics and Maternal-Fetal Medicine, Division of Intramural Research, Eunice Kennedy Shriver National Institute of Child Health and Human Development, National Institutes of Health, U.S. Department of Health and Human Services (NICHD/NIH/DHHS). This study was also, in part, supported with Federal funds from NICHD/NIH/DHHS under Contract No. HHSN275201300006C. Dr. Gomez-Lopez is also supported by the Perinatal Initiative of the Wayne State University School of Medicine.

## Conflict of Interest

The authors declare that the research was conducted in the absence of any commercial or financial relationships that could be construed as a potential conflict of interest.

## Publisher’s Note

All claims expressed in this article are solely those of the authors and do not necessarily represent those of their affiliated organizations, or those of the publisher, the editors and the reviewers. Any product that may be evaluated in this article, or claim that may be made by its manufacturer, is not guaranteed or endorsed by the publisher.
