# Supplementary material for: Dynamic Changes in Uterine NK Cell Subset Frequency and Function Over the Menstrual Cycle and Pregnancy
Source: Front Immunol. 2022 Jun 16;13:880438. doi: 10.3389/fimmu.2022.880438 (PMC9245422; doi:10.3389/fimmu.2022.880438)
Supplement: Supplementary file 1 [file DataSheet_1.pdf]

Whettlock, Woon et al - Supplementary Material

Supplementary Table 1. Demographics from non-pregnant participants

Patient	Age	Parity	Cycle day	Cycle length	Serum progesterone (ng/mL)	Phase assigned	Assessment
1	38	Multiparous	1	28	<1	Menstrual	Phenotype
2	36	Multiparous	1	28	3	Menstrual	Phenotype and function
3	23	Primiparous	2	28	2	Menstrual	Phenotype and function
4	25	Primiparous	1	28	<1	Menstrual	Phenotype
5	21	Primiparous	5	28	1	Menstrual	Phenotype and function
6	26	Primiparous	2	24	Not taken	Menstrual	Phenotype and function
7	38	Multiparous	1	32	1	Menstrual	Phenotype and function
9	24	Primiparous	2	30	1	Menstrual	Phenotype
10	29	Primiparous	10	35	6	Proliferative	Phenotype
11	29	Primiparous	7	28	<1	Proliferative	Phenotype and function
12	35	Multiparous	8	30	4	Proliferative	Phenotype
13	21	Primiparous	10	28	Not taken	Proliferative	Phenotype and function
14	31	Multiparous	10	28	5	Proliferative	Phenotype and function
15	25	Primiparous	11	28	<1	Proliferative	Phenotype and function

16	34	Primiparous	13	28	Not taken	Proliferative	Phenotype and function
17	27	Primiparous	21	30	30	Secretory	Phenotype
18	28	Primiparous	20	21	2	Secretory	Phenotype
19	27	Primiparous	17	27	11	Secretory	Phenotype and function
20	20	Primiparous	15	28	7	Secretory	Phenotype and function
21	30	Primiparous	19	28	51	Secretory	Phenotype and function
22	25	Primiparous	17	28	Not taken	Secretory	Phenotype and function
23	33	Multiparous	19	30	22	Secretory	Phenotype and function
24	40	Multiparous	21	28	28	Secretory	Phenotype and function
25	32	Primiparous	17	28	13	Secretory	Phenotype and function
26	27	Primiparous	20	28	23	Secretory	Phenotype and function
27	27	Primiparous	20	42	7	Secretory	Function
28	44	Multiparous	N/A	N/A	<1	Postpartum 16 weeks	Phenotype
29	35	Multiparous	N/A	N/A	<1	Postpartum 10 weeks	Phenotype
30	37	Multiparous	N/A	N/A	3	Postpartum 4 weeks	Phenotype
31	29	Multiparous	N/A	N/A	<1	Postpartum 6 weeks	Phenotype

Supplementary Table 2. Demographics from pregnant participants (first and third trimester)

Patient	Age	Self-reported Ethnicity	Gravida and Parity	Gestation	Phase	Reason for Caesarean Section	Assessment
1	17	Asian	G2P1	11+2	1T	N/A	Phenotype only
2	24	White	G2P1	8+ 6	1T	N/A	Phenotype and function
3	40	White	G6P4	9+1	1T	N/A	Phenotype and function
4	20	White	G1P0	6+6	1T	N/A	Phenotype and function
5	32	White	G3P0	10+4	1T	N/A	Phenotype and function
6	25	Asian	G1P0	11+5	1T	N/A	Phenotype and function
7	22	White	G1P0	8+3	1T	N/A	Phenotype and function
8	21	White	G3P0	13+0	1T	N/A	Phenotype and function
9	33	White	G3P1	12+6	1T	N/A	Phenotype and function
10	40	Asian	G5P4	8+6	1T	N/A	Phenotype and function
11	40	White	G4P3	10+4	1T	N/A	Function only
12	33	White - Canadian	G2P1	39+0	TNL	Previous CS	Phenotype and function
13	39	Middle Eastern - Turkish	G3P2	38 + 0	TNL	Previous CS	Phenotype and function
14	36	White British	G5P1	37 + 0	TNL	Maternal request	Phenotype and function
15	39	White - Italian	G4P2	38+6	TNL	Previous CS	Phenotype and function
16	37	White - French	G2P1	38 + 0	TNL	Previous CS	Phenotype and function
17	35	White - British	G4P1	39 + 0	TNL	Previous CS	Phenotype and function
18	37	White - Polish	G3P1	39+1	TNL	Previous CS	Phenotype and function
19	33	White British	G1P0	39+0	TNL	Fetal medical condition	Phenotype and function
20	40	White – Eastern European	G2P1	39+3	TNL	Previous CS	Phenotype and function
21	34	White - Greek	G1P0	39+0	TNL	Breech	Phenotype and function
22	33	White - British	G6P0	39+1	TNL	Maternal request	Phenotype and function
23	34	East African - Ethiopian	G3P2	39+4	TNL	Previous CS	Phenotype and function
24	31	White - British	G2P1	39+2	TNL	Breech	Phenotype and function
25	35	White - British	G5P4	39+2	TNL	Previous CS	Phenotype and function
26	31	White - British	G3P1	37+1	TNL	Maternal request	Phenotype and function
27	33	Iranian	G2P1	39+1	TNL	Previous CS	Phenotype and function

28	31	White – British	G6P1	39 + 3	TEaL	Maternal request	Phenotype and function
29	38	Asian – Pakistani	G1P0	38+3	TEaL	Maternal request	Phenotype and function
30	35	White – British	G2P1	39+2	TEaL	Maternal request	Phenotype and function
31	33	White – United States	G2P0	41+1	TEaL	Breech	Phenotype and function
32	29	Mixed race – White and South African	G2P0	40+5	TEaL	Fetal distress	Phenotype and function
33	34	White – British	G1P0	38+3	TEaL	Maternal request	Phenotype and function
34	32	White – United States	G1P0	38+5	TEaL	Maternal request	Phenotype and function
35	36	Mixed race - Brazilian	G3P0	41+0	TEaL	Previous CS	Phenotype and function
36	35	Asian - Indian	G3P0	39+2	TEaL	Maternal request	Phenotype and function
37	37	White - British	G1P0	40+2	TLV	N/A	Phenotype and function
38	31	White - Irish	G1P0	39+5	TLV	N/A	Phenotype and function
39	37	Mixed Asian	G3P1	40+1	TLV	N/A	Phenotype and function
40	31	South European – Italian	G1P0	40+6	TLV	N/A	Phenotype and function
41	29	White - British	G1P0	39+6	TLV	N/A	Phenotype and function
42	31	White - British	G1P0	41+5	TLV	N/A	Phenotype and function
43	34	Caucasian - Czech	G2P1	40+4	TLV	N/A	Phenotype and function
44	36	Asian – Chinese Malaysian	G3P1	40+3	TLV	N/A	Phenotype and function
45	36	Lithuanian	G2P1	39+5	TLV	N/A	Phenotype and function

1T, first trimester; TNL term non labour; TEaL, term in early labour; TLV, term labour vaginal; CS = caesarean section

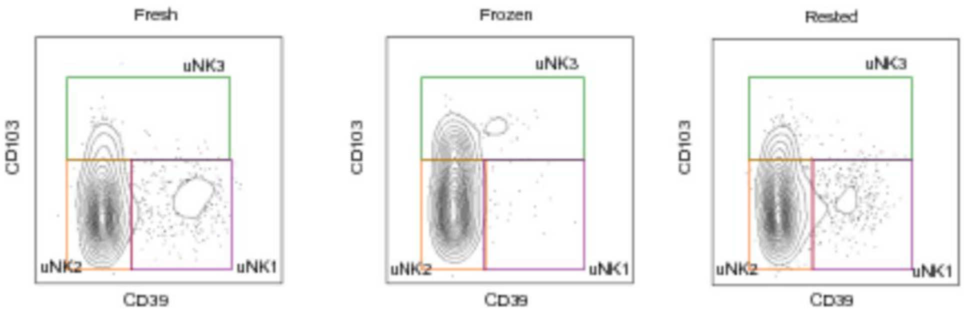

Supplementary Figure 1. Comparison of uNK from first trimester pregnancy, stained and acquired fresh, after cryopreservation and recovery, or after 16 hours rest. All treatments are from the same participant.

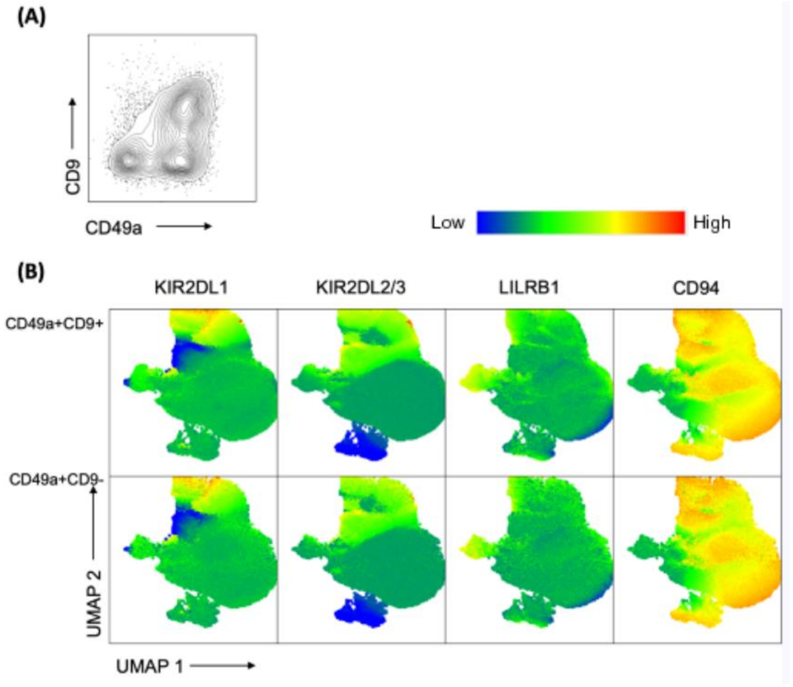

Supplementary Figure 2. (A) Representative staining of CD49a versus CD9 on CD56+CD3- live lymphocytes. (B) CD49a+CD56+CD3- live lymphocytes were concatenated from endometrial, first trimester and third trimester samples. They were gated as CD49a+ CD9+ and CD49a+ CD9- and the expression of the phenotypic markers (KIR23DL1, KIR2DL2/3, LILIRB1 and CD94) was visualized as a heatmap using UMAP plots.

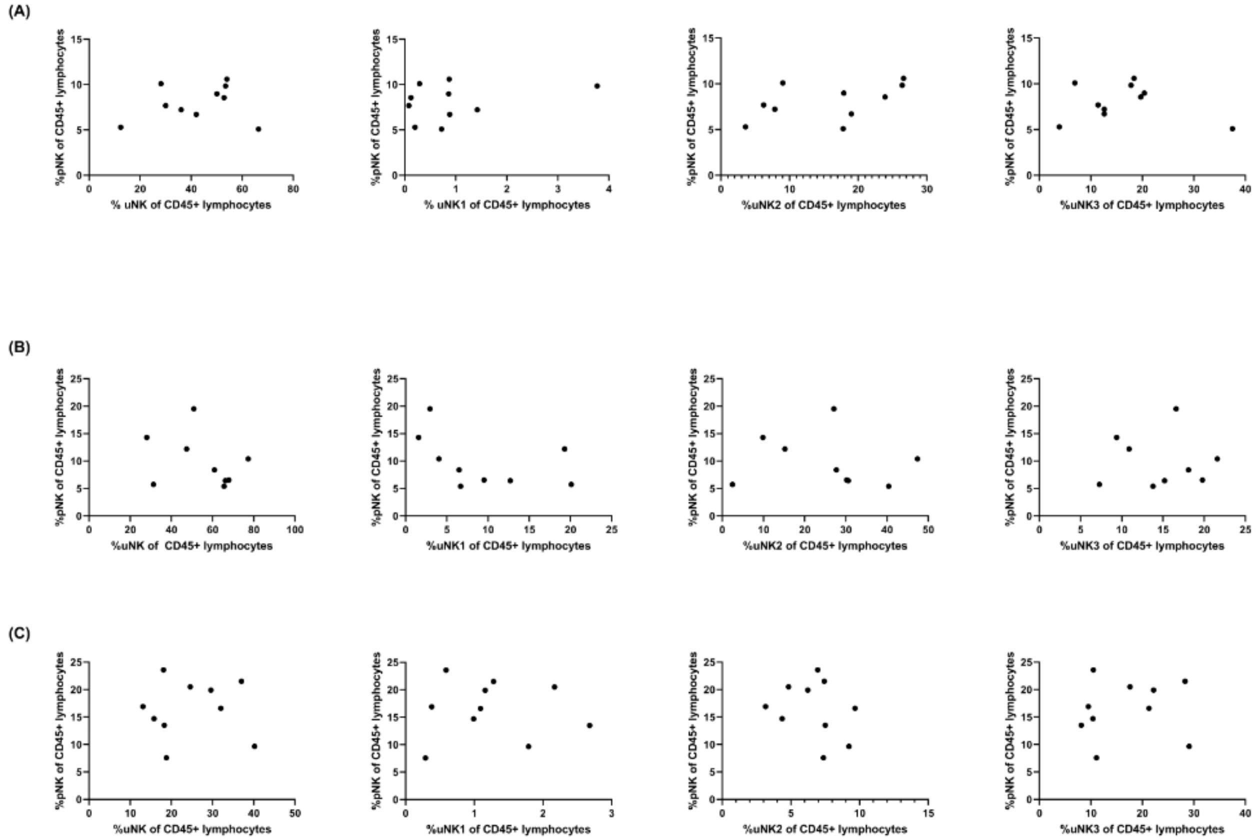

Supplementary Figure 3. Correlation between peripheral and uterine NK cells in secretory phase of the menstrual cycle (A), first trimester (B) and third trimester (C). No significant correlation was found between pNK and uNK, uNK1, uNK2 and uNK3. Additionally, no correlation between CD56^{bright} and -^{dim} and uNK1, uNK2 and uNK3 was found in all phases above when expressed as a percentage of CD45+ lymphocytes (not shown). Statistical testing was done by Spearman's rank correlation.

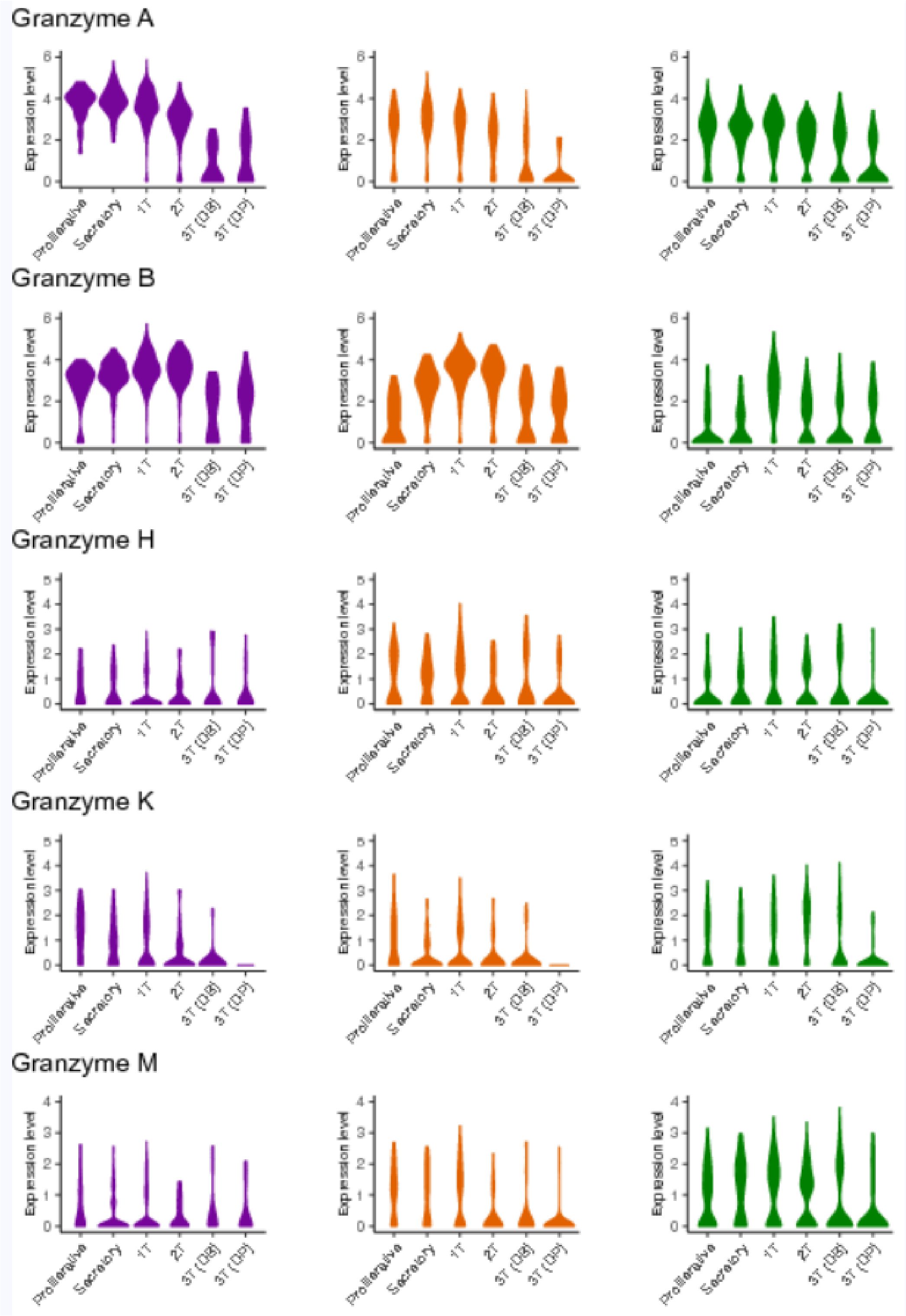

Supplementary Figure 4. Violin plots showing mRNA expression of the three uNK subsets (uNK1 in purple, uNK2 in orange, uNK3 in green) over six phases of the reproductive cycle.

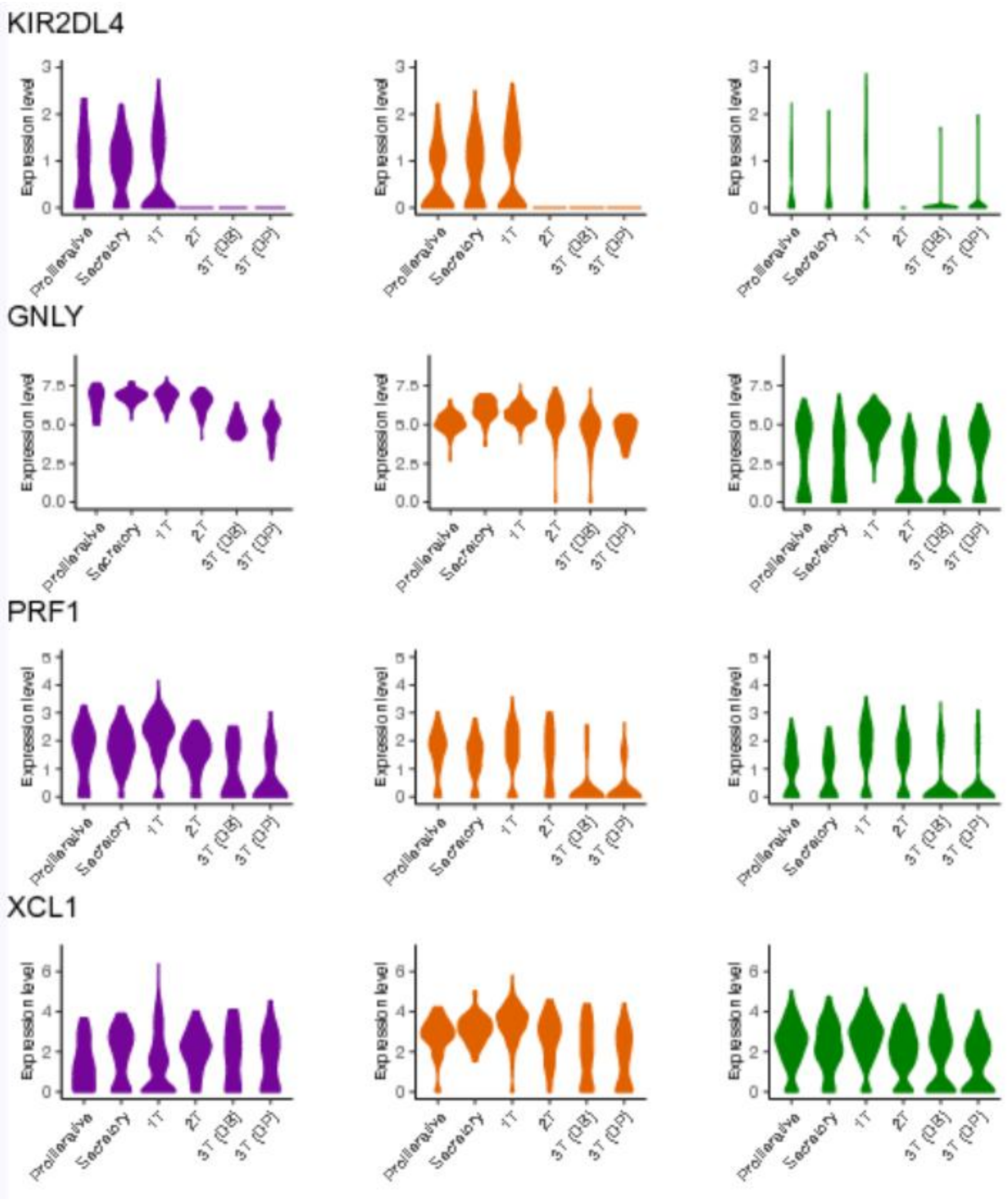

Supplementary Figure 5. Violin plots showing mRNA expression of additional markers of interest in the three uNK subsets (uNK1 in purple, uNK2 in orange, uNK3 in green) across six phases of the reproductive cycle.

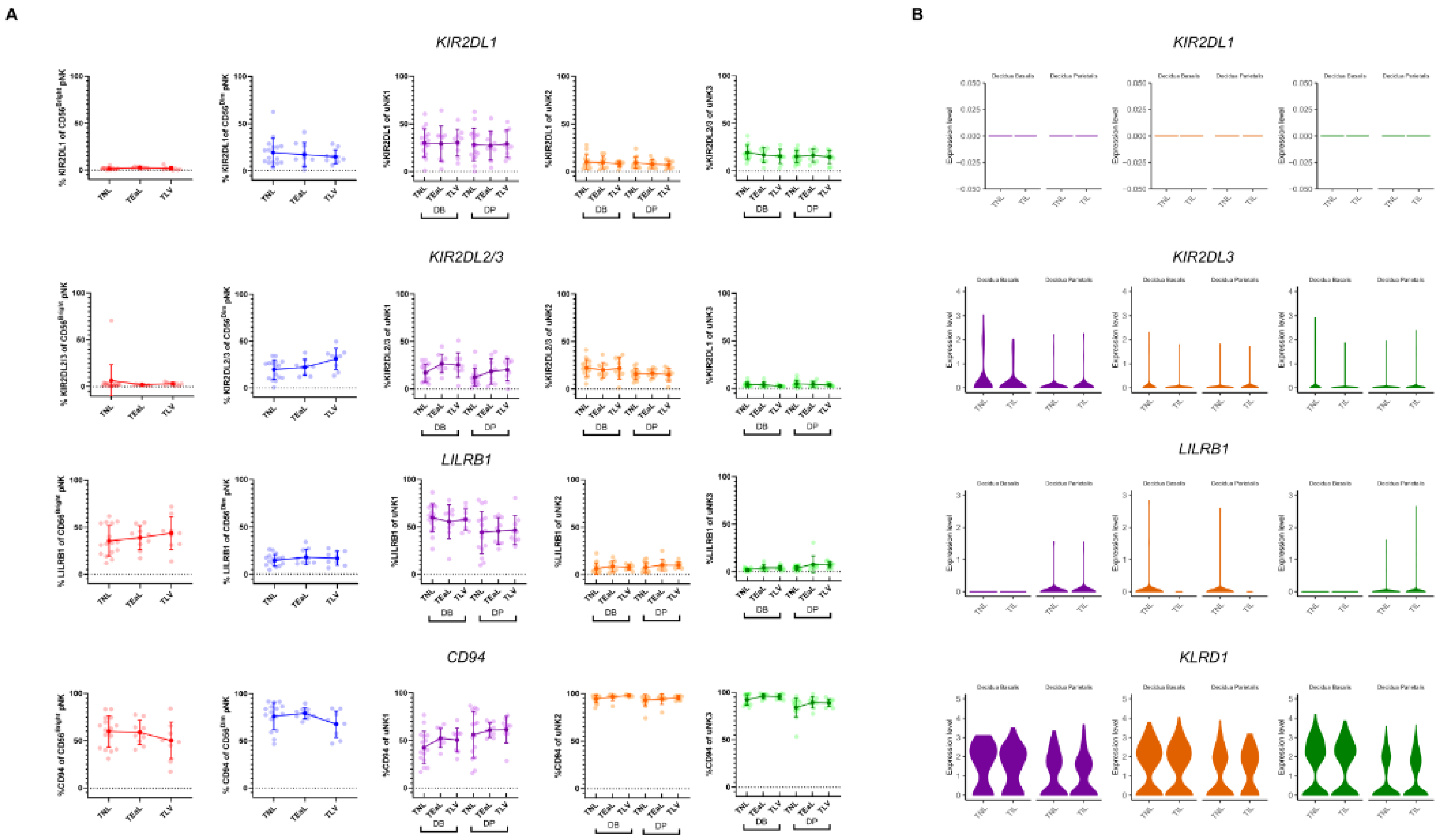

Supplementary Figure 6. Phenotype of NK cells in labour. (A) Graphs showing phenotypes of pNK and uNK in third trimester, acquired by flow cytometry. (B) Violin plots showing corresponding mRNA expression of receptors on uNK, acquired by scRNAseq. TNL, term not in labour; TEaL, term early labour; TLV, term in labour (vaginal); DB, decidua basalis; DP; decidua parietalis.

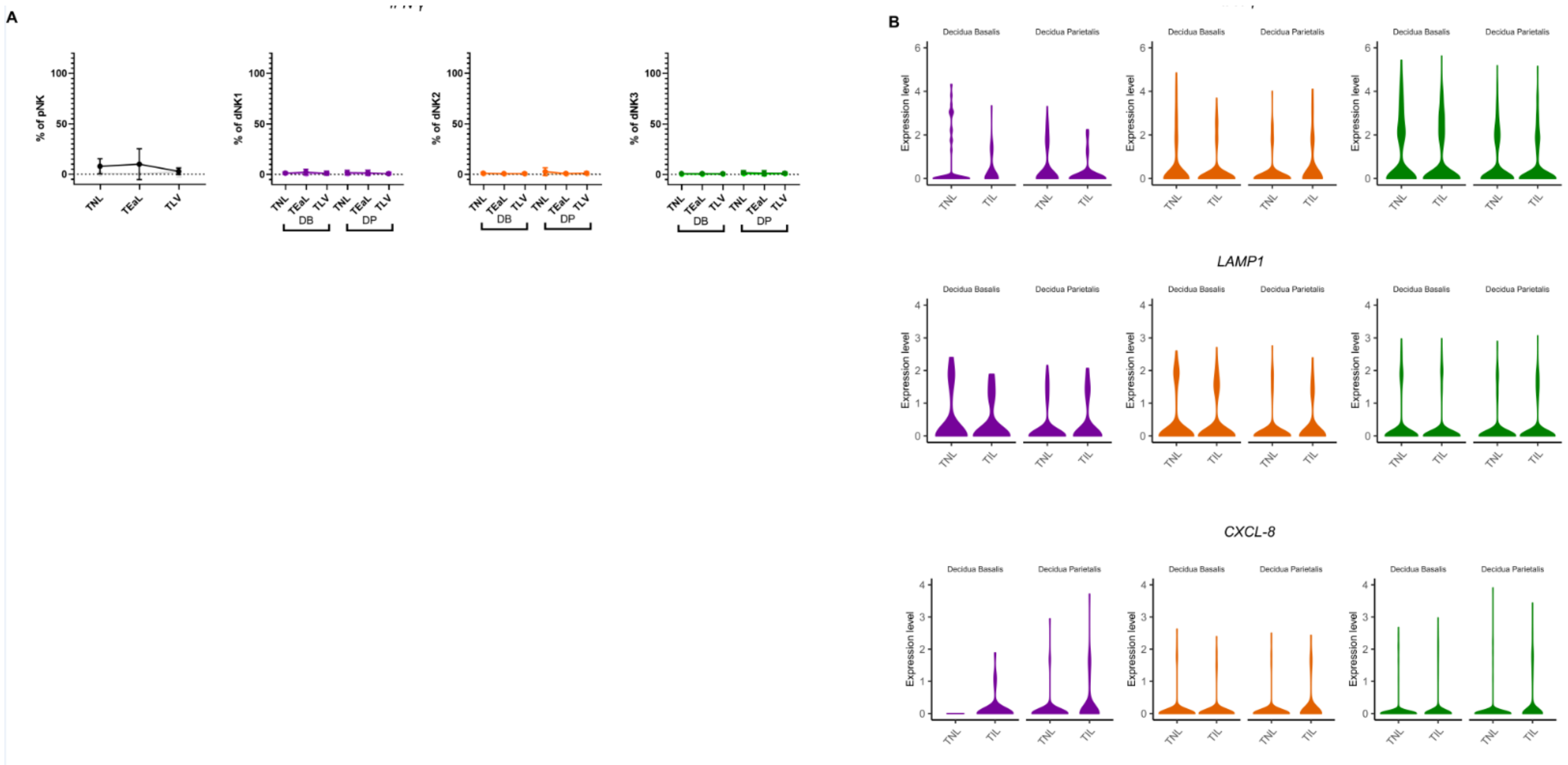

Supplementary Figure 7. Function of NK cells in labour. (A) Graphs showing frequencies of IFNγ in pNK and uNK acquired by flow cytometry in bulk uNK (black), uNK1 (purple), uNK2 (orange) and uNK3 (green). Unstimulated cells are represented by lighter lines and stimulated cells by darker lines. (B) Violin plots showing mRNA expression of IFNG, LAMP1 and CXCL8 in uNK1 (purple), uNK2 (orange) and uNK3 (green). TNL, term not in labour; TEaL, term early labour; TLV, term in labour (vaginal); TIL, term in labour.

Supplementary Table 3 contains a large amount of data and is provided as a separate Excel file.
